# The providers of health services in Lebanon: a survey of physicians

**DOI:** 10.1186/1478-4491-4-4

**Published:** 2006-02-17

**Authors:** Kassem M Kassak, Hassan MK Ghomrawi, Arabia Mohamad Ali Osseiran, Hanaa Kobeissi

**Affiliations:** 1 Department of Health Management and Policy, Faculty of Health Sciences, American University of Beirut, Beirut, Lebanon; 2 Division of Health Services Research & Policy, School of Public Health, University of Minnesota, Minneapolis, Minnesota, USA

## Abstract

**Background:**

Emerging from civil distress carries with it major challenges to reforming a health system. One such challenge is to ensure an adequate supply of competent human resources. The objective of this study was to assess the supply of physicians in Lebanon in 1998, with an assessment of their practice patterns and capacity building.

**Methods:**

Lists of members of physician's associations were examined to determine the number of physicians in Lebanon and their geographical distribution. A self-administered survey targeted 388 physicians (5%) randomly stratified by the five regions of Lebanon. Some 377 providers reported information on their demographic profile, practice patterns and development. Further, information on continuing education activities was acquired.

**Results:**

In Lebanon, the overall physician-to-population ratio was 248 per 100, 000, characterized by an evident maldistribution at the intracountry regional level. Physicians worked 38 hours per week examining on average 21 patients per day, with an average time of 30 minutes spent per visit. They also reported spending 11% of their time waiting for patients. Respondents reported a very wide range of income, with 90% earning less than USD 2,000 per month. Moreover, the continuing education profile revealed a total of 43.7 hours per year, similar to that required for board certification in many developed countries. Conference attendance was the dominant continuing education activity (95% of respondents) and consumed most of the time allotted for continuing education, reported at 32 hours per year.

**Discussion and conclusion:**

Various economic indicators point to an oversupply of physicians in Lebanon and a poor allocation of their time for capacity building. Therefore, it is crucial for decision-makers to closely monitor the increasing supply of providers and institute appropriate intervention strategies, taking into consideration appropriate provision of good-quality services and ensuring that continuing education activities are well established, organized and monitored.

## Background

Human resources in the health care services field continue to play a crucial role in shaping the delivery of health care across the globe. Policies to discipline their practice, shape their jurisdictions and decide their reimbursement schemes have never been simple and have always been faced with various political and economic barriers.

Lebanon is a small country situated on the south-eastern side of the Mediterranean Sea with close to 4 million residents. The country has recently emerged from 17 years of civil disturbance (1975–1992) that has left any surveillance statistics in the country struggling with a large margin of error. One such example is the reported physician-to-population ratio. In 1998, one source claimed that there was one physician for every 770 inhabitants (130 per 100, 000 population) [1], while another indicated that there was one for every 420 residents (238 per 100, 000 population) [2]. Both these sources used the same database, but with obviously different assumptions about how many were actually practising and the actual size of the population.

In addition to an imprecise assessment of physician numbers, one economic observation and another geopolitical fact complicate our understanding of the dynamics of physicians practice. From an economic perspective, the supply of physicians has increased significantly after the end of the civil disturbance for three main reasons: physicians returning to practise in their home country; new medical schools opening in the country; and Lebanese students targeting eastern European universities to attain their medical degree (many eastern European medical schools were opened to foreign students only after the breakdown of the Soviet Union). Reports show an annual increase of 500 to 700 physicians [2]. On the other hand, the demand for medical services, especially tertiary care, has grown substantially, resulting in an unsustainable level of expenses, most of which are paid by the Ministry of Public Health (40%) and other public insurers.

From a geopolitical angle, Lebanon is divided into five regions (muhafazat) with observed differences in their urban/rural mix of areas. The small geographical area of the country (some 10 425 km^2^) and the closeness of regions to each other and to Beirut, the capital, allow much cross-practice between regions. But physicians are unequally distributed between two orders (professional associations) that oversee their practice, although all physicians must pass the same government licensure test. The two orders have slightly different rules for membership and benefits. The two orders advise physicians to seek continuing medical education, but do not require them to do so to maintain the licence to practise medicine. One caters for the physicians of North Lebanon, while the other four regions are under the jurisdiction of the second order (Lebanon order of physicians).

Lebanon is sometimes referred to as "the hospital of the Middle East" because of its good health care delivery. To continue to provide good care, Lebanon must put in place sound health workforce policies that take into account all of the above complex picture. A health sector rehabilitation project estimated to cost USD 50 million was initiated in 1995. The project's main focus was to unify the many non-private financing agencies and to restructure the payment system for health care providers (mainly private hospitals). To our knowledge, no special policies were developed to target physicians' licensure, practice and reimbursement.

The cost attributable to human resources is complicated by the product of two components: expenditures attributable to the providers, and the number of such providers. Therefore, any reform related to supply would definitely have its impact on the magnitude of health care expenditures [3,4]. From that perspective, it was crucial to examine the supply of physicians in Lebanon in order to establish a baseline on the profile of providers of care and their practice, and respond to the need as well as their ways and means of improving their knowledge and skills, and hence, their practice. Therefore, this study aimed at examining the supply of physicians residing in Lebanon and assessing their practice patterns and tendency for capacity development.

## Methods

### Supply of physicians in 1998

All physicians who had passed the colloquium test up till the year 1998 and were registered with one of the two orders were included in the universe for our analysis. The list for this study was acquired from the physicians' two orders and included information about the specialty and subspecialty (where applicable) of the physician, practice address and home and work phone numbers. The database was checked to ensure no duplicates in memberships. To calculate the physician-to-population ratio in 1998, we used the World Health Organization estimate of Lebanon's population: 3.2 million. This figure represents the best available estimate of the population size, as no national population census has been conducted since 1932.

### Practice patterns, reimbursement and continuing education

The sample for this study (n = 388) represented 5% of the general population of registered physicians, randomly selected and stratified by the five major regions (muhafazat) of the country (Table 1). After a phone appointment, data collectors provided the physician with a self-administered questionnaire that was completed while the data collector was present or picked up by the data collector on a later convenient day.

**Table 1:** Distribution of physicians among regions in Lebanon

**Region**	**Population distribution**	**Physicians per 100 000 population**	**%**	**Sample Distribution**	**%**
Beirut	2545	624.7	33%	133	35%
Mount Lebanon	2711	236.7	35%	129	34%
South	879	180.0	11%	45	12%
North	1027	153.1	13%	41	11%
Bekaa	564	141.0	7%	29	8%
National Density	*7726*	248.0	*100%*	377	100%

The self-administered questionnaire was designed to include questions on demographic characteristics, educational background, specialty, job characteristics and activities, and practice setting. In addition, the physicians were asked about their workload: work hours, number of work sites, and proportion of time spent in clinical, managerial, educational and scientific activities, including literature appraisal and research, and time spent for their private life away from their professional practice. Moreover, a subsection was devoted to inquiring about continuing education (CE) activities. Continuing education included activities such as conferences, university CE, non-university CE, meetings of scientific societies, reading scientific journals and audiovisual sessions.

To ensure clarity and comprehensiveness, the questionnaire was face-validated within a group of health services researchers and it was pilot-tested with a sample of residents from the school of medicine at the American University of Beirut. As there is no official classification of the distribution as to rural or urban areas, a panel of experts was asked to classify the site of practice by districts (qazas) into urban or rural. The index was used to investigate if urbanization has an effect on practice style in a small country where areas are in close proximity.

Data were entered and analysed using SPSS 11.5 software. Frequency distributions were first explored, and afterwards chi-square and student t-tests were used to test associations between variables.

## Results

### Supply of physicians in 1998

This study validated that the supply of physicians according to the orders' lists was 7726 physicians registered with the two professional associations for the year 1998, thus yielding an average of 248 physicians per 100, 000 population. Furthermore, the density of practitioners ranged from as high as 625 physicians per 100, 000 population in Beirut, the capital, to as low as 141 physicians per 100 000 population in the Bekaa – generally classified as a rural region (muhafaza) (Table 1). Of the 388 physicians sought for the study, 377 physicians responded to the questionnaire. However, data were not complete on all items on the questionnaires, which explains the missing cases in some of the results.

### Provider characteristics

The demographic profile of the sample was characterized by a higher proportion of males (84%), with an average age of 42.5 years and an average of 13 years of experience. Slightly above 75% of the respondents were between 30 and 49 years of age.

In general, 49% of physicians practised in urban districts (qazas). Though of all the 44% who were general practitioners (which included general medicine, internal medicine, paediatrics, and obstetrics and gynaecology), it was observed that a great proportion (57%) was practising in rural areas (X^2 ^= 4.71, P-value = 0.029). In addition, most of the female physicians in our sample were general practitioners (Table 2). On the other hand, 2.4% were not practising medicine but instead were holding administrative posts in health-related fields.

**Table 2:** Distribution of specialties by sex and degree of urbanization (%)

		**Degree of ubanization**		
Sex		Urban	Rural	Total

Male	GP	56 (34.4)	69 (44.5)	125
	Non-GPs	107 (65.6)	86 (55.5)	193
	**Sub-Total**	**163**	**155**	**318 (84.4%)**
Female	GP	15 (68.2)	26 (70.3)	41
	Non-GPs	7 (31.8)	11 (29.7)	18
	**Sub-Total**	**22**	**37**	**59 (15.6%)**
	**Total**	**185**	**192**	**377**

Physicians residing in Lebanon form a mosaic of educational background and expertise acquired in different parts of the world. A greater proportion of physicians graduated from western European medical schools (31.5%), followed by Lebanese medical schools (29.6%), while only 2.6% graduated from medical schools in the United States and Canada (Table 3).

**Table 3:** Distribution of physicians according to region from which they graduated

**Region**	**Frequency**	**Percentage**
Eastern Europe	69	18.3
Western Europe	119	31.5
North America	10	2.6
Lebanon	112	29.6
MENA countries	11	2.9
Others	5	1.3
No response	51	13.8

**Total**	**377**	**100**

### Practice characteristics

Of the practising physicians, 288 respondents practised mainly in clinics, while the rest practised in hospital-based settings. Of the 288 physicians practising in clinics, 91% reported that they owned their clinic, while only 3.5% reported being hired staff at the practice.

Respondents worked on average 38 hours per week examining on average 21 patients, and spending an average time of 30 minutes per visit (Table 4). Moreover, 189 physicians had contracts with hospitals and spent, on average, over 26 hours per week in contracted hospitals. Physicians estimated that they spent 39% of their time at work examining patients. In addition, they spent almost 11% of their time waiting for patients, and only 3.3% was spent on administrative work (Table 5). Gender differences were observed: female physicians spent significantly less time waiting for patients than did male physicians (7% for females versus 12% for males; P-value = 0.038) and significantly more time is spent in their private life (42% for females versus 34% for males; P-value = 0.007).

**Table 4:** Work activity

	**Mean**	**Standard deviation**
Days worked per week	5.35	0.71
Weeks worked per year	49.76	2.94
Hours worked per week	37.54	9.48
Patients seen per week	21.13	18.04
Time spent per patient examination (min)	30.68	20.29

**Table 5:** Time distribution at work

	**Mean**	**Standard deviation**
% spent examining patients	39.11	17.41
% laboratory work	7.90	12.40
% administrative work	3.28	8.61
% waiting for patients	11.06	15.63
% cancelled appointments	3.55	4.70
% spent on other activities	34.09	20.74

### Financing characteristics

With the presence of different financing agencies, physicians were reimbursed for 54% of their services from private patients (out of pocket) and 27% from privately insured patients, while the Ministry of Public Health contributed 4.8% to the physician's source of income (Table 6). For those who volunteered information on their monthly income (78.2 %), the range was from LBP 200, 000 (about USD 133) to LBP 30 million (about USD 19 ,900), with an average of LBP 2.81 million (USD 1873) and a median of LBP 2 million (about USD 1,300). Slightly above 90% of the respondents estimated their monthly income to be equal to or less than LBP 3 million (about USD 2,000). Finally, those graduating from North American institutions earned significantly higher incomes, when compared to graduates of other regions of the world (Table 7). It is worth mentioning here that the difference in physicians' income between rural and urban settings did not reach statistical significance.

**Table 6:** Sources of financing for physician services

	**Mean**	**Standard deviation**
% Out of pocket	53.52	29.78
% Private Insurance	26.60	26.99
% Public insurance, excluding MOH	5.85	13.45
% Ministry of Health	4.83	14.66
% NSSF	8.88	12.61

**Table 7:** Average monthly income of graduates by region of education, in Lebanese lira (USD 1 ≈ LBP 1500)

	**Mean**	**Standard deviation**
Lebanon	2 810 714.30	3 795 014.68
West Europe	2 984 803.90	4 061 365.85
The Americas	5 625 000.00	8 602 882.27
East Europe	2 770 535.70	4 391 828.95
MENA countries	2 010 000.00	693 541.64
**Entire sample**	**2 933 620.70**	**4 192 710.64**

### Continuing education activities

Close to 90% of the physicians had sought CE activities during the year prior to the study. Most of the respondents reported that attending conferences was the main CE activity (97% of all those attending CE), while reading scientific journals (22.4% of all those attending CE) was of lesser importance. Moreover, the respondents spent an average time of 43.7 hours/year on CE. Of those, 32 hours/year were spent in attending conferences, while 3.15 hours/year were spent on reading scientific journals (Table 8). Furthermore, subjects perceived several barriers to attending CE programs. Lack of time was a major obstacle, reported by 32% of the respondents, while accessibility (16%) and high cost (7%) were of less importance. In addition, private life was a barrier to 7% of the respondents. On the other hand, 47% were satisfied with their present activities and perceived no barriers to participating in CE activities.

**Table 8:** Time, in hours, annually spent on continuing education activities

	**Mean**	**Standard deviation**
Conferences	32.31	21.99
University continuing education	2.41	17.86
Non-university continuing education	1.75	5.20
Scientific societies	2.02	7.39
Reading scientific journals	3.15	8.00
Audiovisuals	2.41	9.93
Other CE activities	0.22	2.05

## Discussion

Human resources play a crucial role in the development and the successful implementation of a health services delivery system. Of importance is their dynamics as a labour force in the provision of health services, their education and continuing education in order to provide good-quality services, and the appropriate use of their services. In spite of its limitations, this descriptive study provides an assessment of physicians' practice patterns and continuing education activities on a national scale.

### The supply

Currently the ratio of physicians to population in Lebanon is one of the highest in the Eastern Mediterranean region. A ratio of 248 physicians per 100,000 population is rather close to figures reported in the United States and OECD countries (Table 9). A number of indicators suggest that the current physician/population ratio represents an oversupply of physicians in Lebanon.

**Table 9:** Supply of physicians in Lebanon compared to other countries

**Country**	**Physicians per 100 000 population**
Germany	319
France	280
**Lebanon**	**248**
United States	245
Egypt	202
Japan	177
Saudi Arabia	166
Jordan	95.5
Turkey	61.0

A change in government regulations or a change in market conditions could lead to a surplus in human resources [6]. Stability in Lebanon after a long period of civil disturbance induced the return of physicians practising abroad, as well as the opening of new medical schools in the country. As a result of this change in market conditions, the supply of physicians grew in a manner that is disproportionately larger than market demand for their services.

This suspected surplus is further complicated by an uneven geographical distribution of practitioners. The physician-per-population ratio greatly varied between regions (muhafazat). Beirut, its suburbs and the Mount Lebanon region (the region closest to Beirut) drained the bulk of physicians.

### Practice patterns

One indication of surplus is when physicians are making a below-normal "rate of return" [6]. In reference to the study, with few exceptions, most of the respondents (90%) earned below USD 2,000 a month. For many of these providers, this monthly income is close to the average cost of living (about USD 1,100 per month) [5] and is intuitively below the expected rate of return on their investment in medical training. We also noticed a deviation in physicians' practice patterns from general norms. Physicians in this study spent almost 11% of their time waiting for patients. This strongly suggests that the physician market has surpassed its saturation point. This matter is further exacerbated by the overhead costs that a solo practice must meet to function properly and effectively. As was indicated earlier, close to 90% are in solo practice and this has major implications for the efficiency and productivity of the practice, which calls for further research to substantiate this impact.

Furthermore, in the early 1990s, Kronfol et al. indicated that after the war ended, there was an influx of medical graduates from the eastern European countries, resulting in a change in the ratio of graduates from different parts of the world, and warned that these would greatly affect the quality of care in Lebanon [7]. It is commonly believed that providers who were trained in more educationally advanced schools were exposed to more technologically advanced medical practice and, hence, tend to comply with such practice standards. On the other hand, those coming from poorer schools are probably less exposed to advanced techniques and may thus have excluded themselves from practising in technologically competitive service delivery settings (i.e. urban settings). We expected that the diversity of providers would have led to segmentation in the health services provided, in terms of type and quality.

It was postulated that graduates of schools in the Americas and western Europe would be more concentrated in the urban areas and graduates from schools in less-developed and developing countries would locate in less urbanized and rural areas. Contrary to expectations, these graduates were almost evenly distributed between urban and rural areas. The very few differences in practice patterns between rural and urban areas may be explained by the absence of any geographical barriers that would induce major differences in the practice patterns of physicians. The country's small geographical area and the relative ease of transportation make it possible for physicians to maintain strong contacts with leading medical institutions in the country and be able to refer their patients to these institutions, no matter how remotely they are located.

### Gender differences in practice patterns

In this study, we found significant differences in the choice of specialty and practice patterns between men and women. Women in Lebanon still tend to avoid specialties that are technically and physically demanding in nature and culturally unfavourable. Most of the female physicians in this sample were GPs and very few (only two) were surgeons. This finding is in accordance with the trends reported in the many European countries [8].

In terms of practice patterns, female physicians were similar to their male counterparts with regard to most of the indicators we examined. Their involvement with their private life might force them to restrict their appointment schedule to certain hours of the day, thus spending less time waiting for patients. Further research in this area will enhance our understanding of the role and effectiveness of women in medical practice in Lebanon and the Middle East, given the very few studies in this area.

### Continuing education activities

Continuing medical education forms an integrated activity in a health professional's career and for that it should be well-structured and well-organized. In this study, the total number of hours for which the physicians sought CE per year is comparable to that in the USA and other developed countries. In other countries in the world where CE is not a requirement, studies showed that physicians pursued CE as a signalling strategy to attract patients [9]. As such, one would wonder if the providers were seeking CE for the sake of updating their knowledge or to compete for patients in a situation of suspected surplus. Moreover, the mix of these activities is very different. Doctors seem to attend conferences much more than they pursue other CE activities. For example, physicians spent no more than 3.5 hours per year reading "any type" of scientific journals, while spending 32 hours attending conferences.

The study findings raise concerns about medical CE activities in Lebanon. One concern is that this might support the earlier notion of oversupply of providers, where a proportion of them have plenty of time to spend on conferences, given their economic distress. The other concern is that conference activities are not monitored, which nurtures doubt about the quality of information disseminated in these conferences. For example, it is well known that the pharmaceutical industry has been very active worldwide in marketing its products to physicians and using CE activities (mostly conferences) as one of its marketing tools [10]. No one questions the importance of such activities, but participation in them must be pursued with great caution as they tend to serve as a fertile medium for dissemination of biased information. Therefore, such events should be well-organized, monitored and evaluated in order to reach an acceptable level of scientific rigor, originality and contribution to the base of knowledge. Furthermore, they should be closely monitored to assess the real credit load.

One cannot but notice that other avenues to continuing education were poorly used. In an age where medicine is rapidly advancing, it is essential that physicians, and other human resources for health, keep abreast of the growing health literature in order to provide the best care. Medical curricula have been emphasizing evidence-based medicine as a process of lifelong, self-directed learning in which caring for patients creates the need for clinically important information about diagnosis, prognosis and therapy [11]. To supplement that, the medical societies in Lebanon should promote critical appraisal of the literature as a major source of knowledge, especially among those who have been away from meetings and conferences. This can be done through workshops, seminars and reading materials.

## Conclusion

In conclusion, human resources for health are the key to proper functioning of any health care system, depending on their adequacy and suitability [12]. They help shape the system by determining what services will be consumed and how, where and in what quantity. Thereby, human resources structure the cost burden on payers, public or private.

Given the abundance of physicians in Lebanon, this study calls for reform. Limiting the number of physicians entering the market is a key control factor and can be achieved through many regulations, the most important of which is requiring a higher passing grade for the colloquium exam. Another strategy is to limit the number of students admitted to the Lebanese medical schools, thus restricting entry to the physicians' market. Providing incentives for individuals to seek and practise other health professions (e.g. nursing) may be another key factor in resolving the problem.

Policy-makers must pay more attention to other health professionals, their supply and practice patterns. While physicians are key players in shaping the medical system, of equal importance is the availability and practice style of other health professionals, such as nurses. They help deliver care at different levels within health systems [13]. At present, around 3,000 nurses are practising in Lebanon. This number includes both registered and practical nurses. The shortage of nursing personnel, the absence of specialization in nursing practice and the lack of delegation of authority to nurses channels their supply mainly to hospital settings where they are needed the most, further shifting most of the services, more specifically primary care, to the side of the physician. This calls for major intervention by all stakeholders but more so from decision-makers and professional associations.

Countries recovering from civil distress must be careful in monitoring and evaluating the influx of human resources, as it might lead to new forms of social and economic distress. This will definitely manipulate the supply side of health care delivery, which will affect and be affected by the country's economy. To minimize such distress to the economy as well as to the individual physician, major policies aimed at restructuring the provision of health services and the entry of the professionals into the system should be enacted, or else another migration will emerge, but this time because of a different war – that with the economy and survivorship. Hand-in-hand with the reforming is the issue of licensing and practice standards. The medical profession must critically evaluate its portals of entry and sustained memberships with appropriate quality checks. Continuing education must be obligatory and not just an option.

## Competing interests

The author(s) declare that they have no competing interests.

## Authors' contributions

KMK, HMKG, AMAO and HK have made substantial contributions to conception and design of questionnaire as well as analysis and interpretation of data. KMK, HMKG and AMAO were involved in drafting the manuscript and revising it critically for important intellectual content. All listed authors read and approved the final manuscript.
